# Hand Dysfunction After Intervention via Distal Versus Conventional Transradial Access: A Meta‐Analysis of Randomized Trials

**DOI:** 10.1002/clc.70359

**Published:** 2026-05-28

**Authors:** Fareena Ishtiaq

**Affiliations:** ^1^ Khyber Medical University Peshawar Pakistan

To the Editor,

I read with great interest the recent meta‐analysis by Fan et al. evaluating hand dysfunction after distal versus conventional transradial access for coronary intervention. The authors should be commended for addressing an important yet underexplored clinical issue, particularly as distal transradial access (dTRA) continues to gain popularity in interventional cardiology. Their effort to synthesize randomized evidence on post‐procedural hand dysfunction is timely and clinically relevant. However, several methodological limitations warrant further discussion [1].

First, the most significant concern is the substantial clinical and methodological heterogeneity among included trials. The authors pooled studies that used markedly different definitions of “hand dysfunction,” ranging from transient numbness and pain to objective grip strength changes and imaging‐detected neuropathy. These outcomes are not clinically equivalent and likely reflect distinct biological mechanisms. Combining them under a single conceptual framework risks oversimplification and may reduce the interpretability of the pooled findings [2].

Second, the principal conclusions regarding hand clumsiness, persistent pain, and postoperative numbness were each derived from only two randomized trials. While statistically significant effect estimates were reported, such limited evidence raises concerns regarding fragility and overprecision. This is particularly important because low study meta‐analyses are highly vulnerable to small study effects and random error, even when formal heterogeneity appears low [3].

Third, outcome assessment tools were highly inconsistent. Some studies used pain‐specific scales such as the Visual Analog Scale and Numeric Rating Scale, while others used the Borg scale, a tool originally developed for perceived exertion rather than pain measurement. Similarly, functional outcomes were assessed using DASH, QuickDASH, physical examination, or ultrasound. Pooling data generated from fundamentally different instruments may compromise construct validity and make direct comparison problematic [2].

Fourth, the review restricted inclusion to English‐language publications, introducing potential language bias. Given the rapid global adoption of dTRA, especially in Asia and Europe, excluding non‐English randomized trials may have omitted relevant data and limited the comprehensiveness of the analysis [3].

Finally, although the article is presented as a meta‐analysis, much of the evidence ultimately relied on narrative synthesis because of inconsistent outcome reporting and variable follow‐up durations. This distinction is important, as readers may otherwise overestimate the certainty and generalizability of the conclusions [3].

While Fan et al. provide an important preliminary overview of hand dysfunction after dTRA versus conventional transradial access, the current evidence remains exploratory rather than definitive. Future studies should prioritize standardized definitions, validated hand function assessment tools, and harmonized follow‐up protocols to allow more reliable pooled estimates and stronger clinical recommendations [2, 3].

## Funding

The author has nothing to report.

## Ethics Statement

The author has nothing to report.

## Consent

The author has nothing to report.

## Transparency Statement

The corresponding author, Fareena Ishtiaq, affirms that this manuscript is an honest, accurate, and transparent account of the study being reported; that no important aspects of the study have been omitted; and that any discrepancies from the study as planned (and, if relevant, registered) have been explained.

## Data Availability

Data sharing is not applicable to this article as no data sets were generated or analyzed during the current study.
